# Phenolic and Non-Polar Fractions of the Extracts from Fruits, Leaves, and Twigs of *Elaeagnus* *rhamnoides* (L.) A. Nelson—The Implications for Human Barrier Cells

**DOI:** 10.3390/molecules25092238

**Published:** 2020-05-09

**Authors:** Beata Sadowska, Joanna Rywaniak, Anna Cichocka, Kinga Cichocka, Jerzy Żuchowski, Urszula Wójcik-Bojek, Marzena Więckowska-Szakiel, Barbara Różalska

**Affiliations:** 1 Department of Immunology and Infectious Biology, Institute of Microbiology, Biotechnology and Immunology, Faculty of Biology and Environmental Protection, University of Lodz, Banacha 12/16, 90-237 Lodz, Poland; joanna.rywaniak@biol.uni.lodz.pl (J.R.); annacichocka95@gmail.com (A.C.); kingacichocka1@gmail.com (K.C.); urszula.wojcik@unilodz.eu (U.W.-B.); marzena.wieckowska@biol.uni.lodz.pl (M.W.-S.); barbara.rozalska@biol.uni.lodz.pl (B.R.); 2 Department of Biochemistry, Institute of Soil Science and Plant Cultivation, State Research Institute, Czartoryskich 8, 24-100 Pulawy, Poland; jzuchowski@iung.pulawy.pl

**Keywords:** Caco-2, *Elaeagnus rhamnoides*, HFF-1, immunomodulation, physiological barrier, plant extracts

## Abstract

Biological potential of plant extracts are widely described. Because their oral or topical administration is usually recommended, intestinal mucous and skin are the first surfaces exposed to such preparations. Therefore, we asked the question whether phenolic and non-polar fractions of the extracts from fruits, twigs, and leaves of sea buckthorn (*Elaeagnus* *rhamnoides* (L.) A. Nelson) would be able to modulate the functions of human physiological barrier. The study was carried on caucasian colon epithelial-like Caco-2 cells and human foreskin fibroblasts HFF-1 line. Cell secretory activity (ELISA), the expression of cell surface molecules (flow cytometry), cell migration during wound healing in vitro (scratch assay) were assessed. It was demonstrated for the first time, that sea buckthorn extracts can improve intestinal and skin barrier by increasing of ICAM-1 expression on colon epithelial cells and intensification of IL-8 production by fibroblasts. On the other hand, an inhibition of fibroblasts migration in the presence of those preparations was noted. Therefore, greater attention should be paid on precise description of plant extracts effect depended on target cells and their role to give adequate recommendations for such preparations use.

## 1. Introduction

Fruits, vegetables, seeds, herbs and any plant-derived products rich in valuable secondary metabolites, such as phenolic compounds with the best known group of flavonoids, are usually recognized as beneficial for human health. Practically from the beginning of *Homo sapiens* existence they have been used not only as a food, but also for a treatment of various ailments within right now so called phytotherapy or herbal medicine. Antioxidative, immunomodulatory, anti-proliferative, anti-cancer, anti-atherosclerotic, anti-platelet (antiaggregative), antimicrobial, antiviral, antiallergic, diuretic, or topically anesthetic activity of plant-origin extracts have been described in the literature [1,2,3,4,5,6]. In the context of presented study their immunomodulatory properties are the most interesting. The ability of plant polyphenols (mainly flavonoids) to modulate the activation of many cell types has been presented in the literature. Their effect on cytokines/chemokines release, such as TNF-α, IL-1β, IL-6, IL-8, IL-10, MCP-1, also reactive oxygen species (ROS) and nitric oxide (NO) production by peripheral blood mononuclear cells and macrophages was demonstrated [3,7,8]. Flavonoids such as apigenin and luteolin were described as able to inhibit inflammation in central nervous system via suppression the activity/release of inducible nitric oxide synthase (iNOS), cyclooxygenase 2 (COX-2), TNF-α IL-1β, IL-6, IL-31, and IL-33 produced by microglia cells [9]. However, since oral (e.g., as infusions, solutions, pellets) or topical (e.g., as creams, ointments, backfills) administration is usually recommended for most of plant extracts containing preparations, intestinal mucous and skin are the first surfaces exposed to such products. Moreover, because of direct contact, a concentration of active compounds will also be the highest in those two niches thus conditioning the strongest effect of the extracts. It is assumed, that most of the polyphenols delivered orally are not absorbed in the small intestine, but they are degraded by gut microbiota to other products (e.g., phenolic acids) in the large intestine [10,11]. For instance, 4-hydroxybenzoic acid and vanillic acid are the products of bacterial metabolism of catechin, epicatechin, epicatechin gallate, and epigallocatechin gallate being the major green tea polyphenols [12]. So the metabolites present in blood and other tissues are different from these primary ingested. Furthermore, the concentrations of these compounds in plasma are usually very low comparing with raw uptake, for instance after single dose of 150–2000 mg total anthocyanins, their plasma concentration has reached only 10–50 nmol/L after mean time 1.5 h [11]. Because of limited plant extracts tissue bioavailability and their metabolic transformation, the study of their pharmacokinetics is quite difficult. However, the most prominent and typical for unchanged components the effects should be expected in gut mucosa and skin after, respectively, oral or topical administration. This can have serious implications when we realize, that both are the barrier organs separating underlying tissues from external environment and controlling alien antigens penetration into the human body [13,14,15,16,17]. Thus their proper functioning as the first line of defense within innate immunity determine at least our health and often also a life.

*Elaeagnus rhamnoides* (L.) A. Nelson, previously known as *Hippophae rhamnoides* (L.) and commonly called sea buckthorn, is a Eurasian scrub with yellow-orange berries being since ancient time a subject of great interest because of their pro-health effect resulting from such valuable biological properties as antioxidative, antiaggregative or antimicrobial activity [4,5,6,18,19]. In our previous studies antimicrobial and cytotoxic activity of phenolic and non-polar fractions of *E*. *rhamnoides* (L.) extracts from fruits, twigs, and leaves, as well as their impact on *Staphylococcus aureus* and *Candida albicans* properties was evaluated and their high anti-virulence potential was demonstrated [20]. Here, we make the hypothesis that fractionated sea buckthorn extracts would also be able to modulate the functions of intestinal and skin barrier. Caucasian colon epithelial-like Caco-2 cells were used as in vitro model of intestinal epithelium and their secretory activity in terms of proinflammatory cytokines, as well as an expression of cell surface adhesive molecules after exposure to tested preparations were assessed. Since dermal fibroblasts and keratinocytes mobilization and proliferation are key elements of wound healing, we applied human foreskin fibroblasts HFF-1 line to determine the effect of fractionated *E*. *rhamnoides* extracts on skin barrier in so called scratch assay simulating wound repair processes. Secretory activity of HFF-1 was also tested.

## 2. Results and Discussion

At present study we focused on *E*. *rhamnoides* (L.) extracts effect on the cells forming physiological barrier of human body, which probably would be the first cells exposed to such preparations after oral or topical application in vivo. The impact of phenolic and non-polar fractions of the extracts from sea buckthorn fruits, twigs, and leaves on Caco-2 cells secretory activity in terms of IL-8 and MIP-1α production, as well as an expression of cell surface ICAM-1 and CD49f (as cell marker) was assessed by ELISA and flow cytometry, respectively. The same ELISA method was used to determine the effect of tested plant extracts on proinflammatory cytokines (IL-8, MIP-1α and TNF-α) production by HFF-1 fibroblasts. Moreover, in vitro scratch assay in HFF-1 culture was prepared to assess the impact of those preparations on wound repair process. Based on previously made phytochemical analysis of fractionated *E*. *rhamnoides* extracts, which results have been already published [20,21] and are briefly presented in Table 1, Table 2 and Table 3, simultaneously the cells were exposed to reference compounds. They were quercetin, (−)-epicatechin, ursolic acid, and ellagic acid, which represented the groups of chemical substances being a dominant components of plant-origin preparations tested.

### 2.1. The Effect of Fractionated E. rhamnoides Extracts and Reference Compounds on Caco-2 Cells

The intestinal mucosa is formed by a single layer of epithelial cells closely linked to each other by so called tight junctions created by transmembrane proteins such as claudins, occludins, and junctional adhesion molecules (JAM), as well as by adherent junctions formed by desmosomes and cadherins connected to the cell cytoskeleton. Epithelial cells are covered by protective mucus composed mainly by mucin 2 and glycans secreted by goblet cells. The correct expression and action of junction proteins, intercellular adhesion molecules and mucus contribute to mucosal homeostasis by controlling translocation of pathogenic microorganisms, allergens, carcinogens and other molecules across the intestinal mucosa. Because of barrier function a lot of immunocompetent cells are also located close to intestinal epithelium forming gut-associated lymphoid tissue (GALT). Macrophages, lymphocytes, and dendritic cells form organized structures named Peyer’s patches covered by follicle-associated epithelium and participate in both intestinal and systemic immunity [15,16,22]. Intercellular cross-talk between epithelial cells and immunocompetent cells is based on both direct contact and secreted signaling molecules, such as cytokines/chemokines. Interleukin 8 (IL-8) belongs to chemokines and it is produced by many cell types, including neutrophils, monocytes, lymphocytes T, fibroblasts, keratinocytes, endothelial cells, and epithelial cells. IL-8 possesses strong proinflammatory activity causing mainly neutrophils chemotaxis, degranulation, CR1 and CR3 expression, neutrophil extracellular traps (NETs) formation, and thus an increase of antimicrobial and cytotoxic activity of these cells. IL-8 acts also on T lymphocytes, NK cells and basophils inducing a secretion of anaphylaxis mediators, such as histamine [23,24].

**Figure 1:**
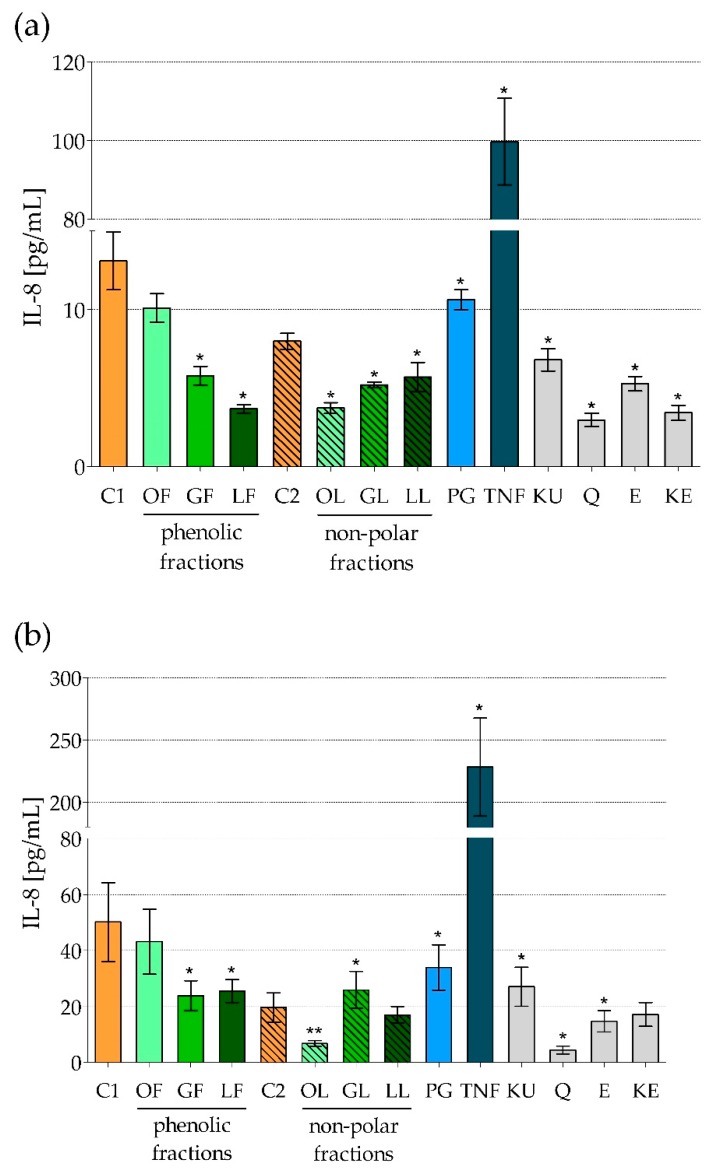
IL-8 production by human colon epithelial-like cells line Caco-2 exposed to fractionated *Elaeagnus rhamnoides* (L.) extracts and reference compounds: (**a**) 6 h; (**b**) 24 h, tested by ELISA. The results are given as a mean concentration (pg/mL) ± standard error. C1—control cells in culture medium with Me-OH, OF/GF/LF—phenolic fractions of fruit/twig/leaf extracts, C2—control cells in culture medium with DMSO, OL/GL/LL—non-polar fractions of fruit/twig/leaf extracts, PG—*Staphylococcus aureus* peptidoglycan, TNF—murine tumor necrosis factor alpha, KU—ursolic acid, Q—quercetin, E—(−)-epicatechin, KE—ellagic acid; * *p* < 0.05, ** *p* < 0.005.

In order to test an effect of *E*. *rhamnoides*-derived products and related compounds, Caco-2 cells were exposed shortly (6 h) or longer (24 h) to: (i) Phenolic and non-polar fractions of the extracts (100 µg/mL); (ii) reference compounds such as quercetin, (−)-epicatechin, ursolic, and ellagic acids (100 µg/mL); (iii) pro-inflammatory molecules such as *S*. *aureus* peptidoglycan (PG; 1 µg/mL) and murine tumor necrosis factor alpha (TNF; 500 ng/mL). Among pro-inflammatory cytokines tested only IL-8 was produced by Caco-2 cells. The absorbance of cell supernatants towards MIP-1α did not exceed established cut-off (data not shown). The results obtained for IL-8 are presented in Figure 1. To make it easier to track the mode of action of tested preparations on Caco-2 cells Table 4 has also been prepared.

As expected, TNF exerted the strongest stimulatory effect on Caco-2 in terms of IL-8 production. Significant rise in IL-8 production was also observed in PG-stimulated cells. While most of sea buckthorn fractionated extracts significantly inhibited secretion of this chemokine (Figure 1, Table 4). After 6 h of cell treatment phenolic fraction of *E*. *rhamnoides* leaf extract (LF) had the strongest inhibitory effect (Figure 1a, Table 4). Similar tendency was observed after long-lasting cells exposure but then non-polar fraction of *E*. *rhamnoides* fruit extract (OL) was the most potent inhibitor (Figure 1b, Table 4). Non-polar fraction of twig extract (GL) was an only exception in mode of natural extracts’ action causing significant increase of IL-8 production after 24-h exposure of the cells (Figure 1b, Table 4).

Interestingly, there is no a huge difference in main chemical composition between hydrophobic fractions of the extracts. Mainly OL and GL, both rich in triterpenoids and acylated triterpenoids and not containing triterpenoid saponins, are similar [20]. Thus, the composition seems not to be an only explanation of different GL activity against Caco-2 cells after their prolonged exposure. On the other hand, GL did not contain quercetin glycosides, which were presented in significant amounts in phenolic fraction of *E*. *rhamnoides* leaf extract (LF) and fruit extract (OF), also in small quantities in non-polar leaf extract (LL) [20,21]. This maybe important since quercetin (Q) possessed the strongest inhibitory activity among all the reference compounds tested (Figure 1, Table 4). Quercetin is one of the most often isolated the flavonoids (flavonol class) from plant materials. A broad spectrum of its biological activity, including antioxidant, anti-inflammatory, anti-aggregative, hypotensive, antihypercholesterolemic, anti-cancer, and antimicrobial properties, is the basis of recommendation to consume quercetin-rich foods for pro-health function [25,26,27,28]. As it was described in the literature anti-inflammatory effect of quercetin results mainly from the ability to direct scavenging of free radicals (anti-oxidative action) and to inhibit the activity of cyclooxygenase 2 and lipoxygenase—the enzymes participating in prostaglandins and leukotrienes formation [25,26,27]. The effect of quercetin on metabolic processes of activated immunocompetent cells, such as pro-inflammatory macrophages M1, particularly its ability to reprogramming of tricarboxylic acids cycle by decreasing succinate and increasing citrate, was proposed as molecular basis for anti-inflammatory activity of this flavonoid [8]. Here we demonstrated strong inhibitory effect against IL-8 production by epithelial cells, which maybe a novel mechanism of quercetin’s anti-inflammatory activity. Michalski et al. [29] showed that quercetin reduced also a production of other pro-inflammatory cytokine IL-12p70 by LPS-stimulated dendritic cells, which resulted in quenching of inflammation.

Anti-inflammatory activity of plant-derived products consisting in repression of IL-8 production was also described by Kim et al. [30] for green tea polyphenols in human nasal fibroblasts. Considering demonstrated inhibitory activity of fractionated *E*. *rhamnoides* extracts (except GL) and the reference compounds in terms of IL-8 production by Caco-2 cells a potential usefulness of these preparations as the products supporting a quenching of inflammation in the intestines can be suggested. Inflammatory bowel disease (IBD)—chronic nonspecific inflammation-associated disorder of gastrointestinal tract seems to be a good candidate for such type of potential supportive therapy. Genetic and environmental factors, intestinal microbiota, as well as immunological abnormalities are considered as predisposing to IBD development, although the exact cause remains still unknown. However, an inflammation associated with massive infiltration of immunocompetent cells into the lamina propria and increased production of proinflammatory cytokines (TNF-α, IL-1β, IFN-γ, the members of IL-23/Th17 pathway group) is characteristic for IBD [31,32]. Previously we did not demonstrate the cytotoxic activity of sea buckthorn phenolic fractions against HFF-1 up to 1 mg/mL for fruit and twig extracts and up to 500 µg/mL for leaf extract [20]. Therefore, phenolic fractions from *E*. *rhamnoides* leaves (LF) or twigs (GF) could be used as probably safe dietary supplements inhibiting chemoattractant production and thus decreasing infiltration of immunocompetent cells to lamina propria. However, this hypothesis needs to be confirmed by the study on animal model of IBD. Moreover, safety use of these extracts is still open problem, until we know their final form and pharmacokinetics. If we assume oral administration like food supplements, sea buckthorn extracts could be applied in lyophilized form as tablets/capsules or could serve to prepare infusions. In both cases the safety of the solvents used in present study (Me-OH or DMSO) no need to be considered, but other questions appear, e.g., about the bioavailability and thus the safe and effective concentrations of the extracts/their components (administered orally and obtained in host tissues). Therefore, the recommendations given here must be treated only as hypothesis.

**Figure 2:**
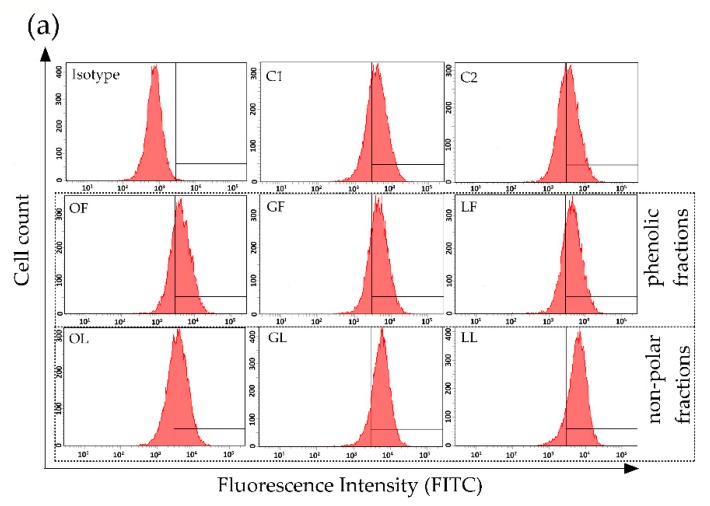

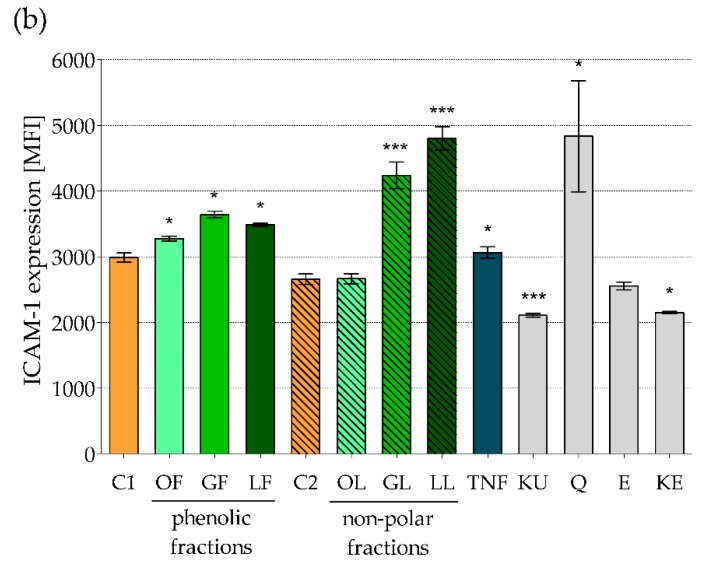
ICAM-1 expression on human colon epithelial-like cells line Caco-2 surface after 24 h exposure to fractionated *E*. *rhamnoides* (L.) extracts and reference compounds, tested by flow cytometry: (**a**) representative histograms; (**b**) quantitative data given as a mean fluorescence units (MFI) ± standard error. C1—control cells in culture medium with Me-OH, OF/GF/LF—phenolic fractions of fruit/twig/leaf extracts, C2—control cells in culture medium with DMSO, OL/GL/LL—non-polar fractions of fruit/twig/leaf extracts, PG—*S. aureus* peptidoglycan, TNF—murine tumor necrosis factor alpha, KU—ursolic acid, Q—quercetin, E—(−)-epicatechin, KE—ellagic acid; * *p* < 0.05, *** *p* < 0.001.

Although catherins and transmembrane tight junction proteins between enterocytes regulate paracellular transport, the barrier function of intestinal mucosa depends on proper functioning of many mechanisms, including also the interactions between epithelial cells and immunocompetent cells. These interactions are mediated by intercellular adhesion molecules such as ICAM-1 and their specific receptors on leukocytes usually from integrin family. ICAM-1 is a ligand for integrin CD11a/CD18 known also as leukocyte function associated antigen 1 (LFA-1) [33]. Therefore, surface expression of ICAM-1 on Caco-2 CD49f-positive cells exposed to fractionated *E*. *rhamnoides* extracts and reference compounds was also assessed. It was demonstrated, that all tested sea buckthorn extracts intensified the expression of ICAM-1 on Caco-2 cells after 24-h exposure to plant material used at a concentration of 100 µg/mL (Figure 2).

The most significant effect was observed after Caco-2 exposure to non-polar fractions from twigs (GL) and leaves (LL) increasing of ICAM-1 expression of 59.4% and 80.5% in comparison to ICAM-1 expression on control C2 cells, respectively (Figure 2b). Interestingly, all sea buckthorn extracts were more potent in regard to this activity than TNF causing an increase of 15.2% only. Based on our results from in vitro study we can speculate that the presence of *E*. *rhamnoides* extracts in the intestines can improve interaction between epithelial cells and leukocytes increasing ICAM-1 expression and thereby accelerate the immune response to pathogens or allergens taken with food. In addition, the improvement of intestinal barrier by sea buckthorn extracts should occur without possible side effects accompanying the classic inflammation involving intensified cell degranulation and pro-inflammatory cytokines production. While reference compounds tested, except quercetin, inhibited an expression of ICAM-1 on Caco-2 cells in the range of 3.9%–20.7% comparing with control cells (Figure 2b). Thus, once again the effect of complex plant extracts did not strictly correlate with an activity of their single components, depending rather on many compounds coexistence in one preparation and possible their synergistic or antagonistic effect, probably even on structure level as was described below. Chen et al. [33] also demonstrated, that such flavonoids as kaempferol, chrysin, apigenin and luteolin effectively inhibited ICAM-1 expression in TNF-induced respiratory epithelial cells. It was suggested that inhibition of kinases’ activity, e.g., mitogen-activated protein kinases (MAPK) and c-Jun NH(2)-terminal kinase (JNK), involving structure-activity relationships, was molecular basis for such anty-ICAM-1 effect [33]. However, in in vivo research still there were no significant differences in plasma ICAM-1 between healthy volunteers consuming large or small amount of vegetables, berries and apples during 6-weeks diet [34]. Interestingly, Ohkita et al. [35] described the inhibitory activity of grape extract prepared from commercially available grape seeds and skin on ICAM-1 gene expression in endothelial cells, which results seem to be opposite to these observed by us for fractionated *E*. *rhamnoides* extracts. But it should be noted, that such inhibitory effect was shown only for the cells stimulated later (after exposure to grape extract) by TNF-α. While ICAM-1 gene expression in unstimulated endothelial cells was even a little bit higher after exposure to grape extract than in control (not exposed to the extract) cells [35]. Thus, in normal conditions (without pro-inflammatory stimuli) grape extract acted similarly to our sea buckthorn extracts.

Summarizing our study on Caco-2 cells, recommendations for use of plant-origin preparations should be strictly dependent on both target cells and health condition of host organism. Most of tested sea buckthorn extracts as inhibitors of IL-8 production could be proposed for oral administration by IBD suffering patients. However, it is worth to pay attention to non-polar fraction of *E*. *rhamnoides* twig extract (GL) as the only one causing intensification of IL-8 production after 24-h cells exposure, which was correlated with significant increase of ICAM-1 expression. A similar observation was also made to non-polar fraction of sea buckthorn leaf extract (LL) being strong ICAM-1 inducer. Thus, oral administration of these two extracts could be recommended for healthy persons as one of natural method to improve of intestinal barrier effectiveness. It is worth noting, that all tested fractions were found to be safe for human fibroblasts and blood platelets [20,36]. Many sea buckthorn-containing products have been approved as food (e.g., fruit juices jams and jelly, seed oils, wine) or food and medicinal supplements (e.g., powdered fruit, leaf infusions and extracts) [6,19,37,38]. However, so far only *E*. *rhamnoides* fruits, seeds and leaf have found a real application in the food, pharmaceutical and cosmetics industry, and there is no information on the use of twigs. For this reason and also because of only in vitro study, our pro-health hypothesis requires confirmation on experimental animal model, both in normal conditions and during intestinal inflammatory disease or food infection.

### 2.2. The Impact of Fractionated E. rhamnoides Extracts and Reference Compounds on HFF-1 Cells

A lot of mechanisms, cell types and the products are involved in skin functioning as main barrier, including exfoliation of dead epidermis layers, secretory activity of keratinocytes, low pH, toxic lipids and free fatty acids forming so called sebum, lysozyme and other antimicrobial peptides. The major “defensive players” also include epidermis-derived alarmins and pro-inflammatory cytokines, antibodies, phagocytes, antigen presenting cells, as well as skin microbiota. However, it seems that maintaining skin continuity is the most prominent, and effective wound healing is key to restoring the skin barrier [13,14,39]. It is well known that wound healing is a multistep process inclusive immediate response, inflammatory, proliferative, migration phases, and finally resolution phase. During first two steps blood platelets are activated, fibrin clot is forming, and leukocytes (mainly neutrophils, then macrophages) are recruited to the wound to remove invading microorganisms, other alien antigens, damaged cells or their components. Proliferative and migration phase is dedicated to reconstruct damage tissue. Fibroblast play in this stage a crucial role migrating, dividing and producing skeleton proteins (e.g., collagen) which leads to the formation of granulation tissue, wound closure and then to dermis reconstruction. The resolution phase is necessary for restoration of skin full functionality. Proliferation of the cells are stopped, leukocytes, cytokines, and other proteins no longer needed are degraded and cleared [40,41,42]. Because of fibroblasts importance in wound healing these cells have become our model for further in vitro study the effect of fractionated *E*. *rhamnoides* extracts on skin barrier.

**Figure 3:**
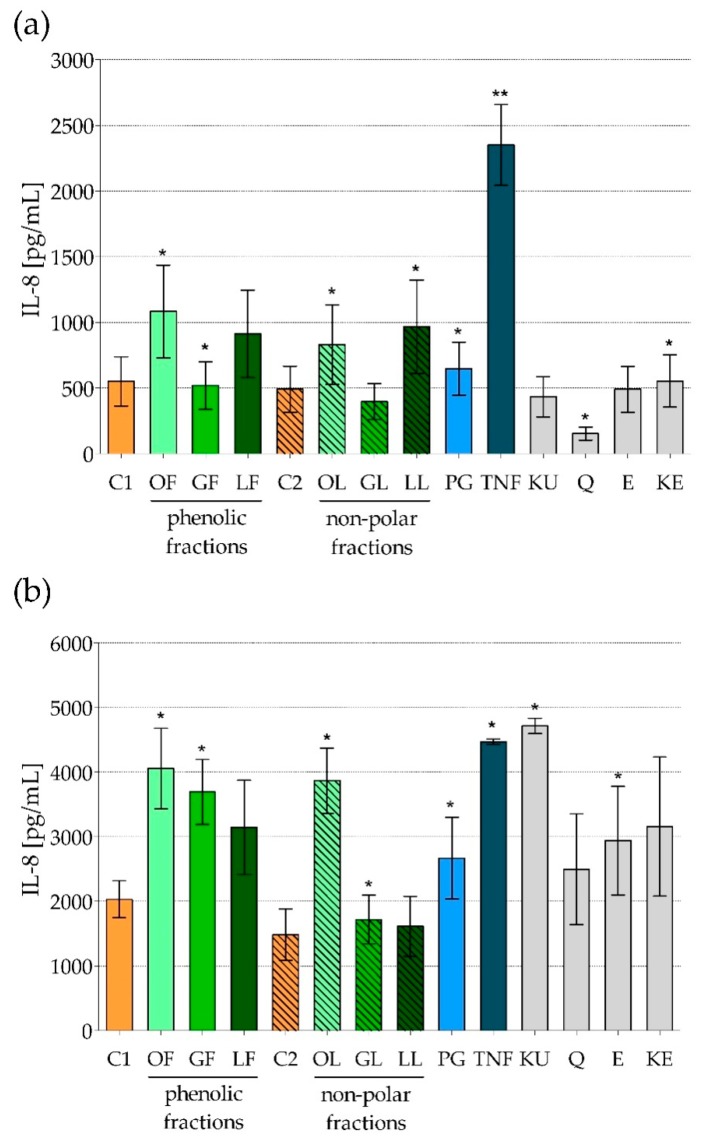
IL-8 production by human foreskin fibroblasts line HFF-1 exposed to fractionated *E*. *rhamnoides* (L.) extracts and reference compounds: (**a**) 6 h; (**b**) 24 h, tested by ELISA. The results are given as a mean concentration [pg/mL] ± standard error. C1—control cells in culture medium with Me-OH, OF/GF/LF—phenolic fractions of fruit/twig/leaf extracts, C2—control cells in culture medium with DMSO, OL/GL/LL—non-polar fractions of fruit/twig/leaf extracts, PG—*S. aureus* peptidoglycan, TNF—murine tumor necrosis factor alpha, KU—ursolic acid, Q—quercetin, E—(−)-epicatechin, KE—ellagic acid; * *p* < 0.05, ** *p* < 0.01.

Proinflammatory cytokines/chemokines such as IL-8, MIP-1α, and TNF-α production by human foreskin fibroblasts line HFF-1 after short (6 h) and long (24 h) exposure to phenolic and non-polar fractions of *E*. *rhamnoides* extracts (100 µg/mL), as well as reference compounds (100 µg/mL) was assessed. The cell stimulators as PG and TNF were used to check cell positive response. The absorbance of cell supernatants tested by ELISA for MIP-1α and TNF-α presence reached just a little over established cut-off, which did not allow for the assess of these cytokines production (data not shown). HFF-1 cells exposed 24 h to TNF were the only exception secreting MIP-1α, which reached the level of 82.8 ± 35.8 pg/mL. While, IL-8 was intensively produced by HFF-1 in response to all above stimuli and obtained results are presented in Figure 3. Just like previously for Caco-2 cells, the tendencies of action of tested preparations on HFF-1 secretory activity has also been shown in Table 5.

First of all, it was demonstrated that the level of IL-8 produced by HFF-1 cells was many times higher than that produced by Caco-2 cells. Considering only control cells a concentration of IL-8 after 6-h co-incubation reached 490.9–550.8 pg/mL in HFF-1 culture, versus 7.9–13.1 pg/mL in Caco-2 culture. Prolonged to 24 h incubation caused of course an increase of absolute values of IL-8 concentration but the differences between both cell types remained similar. Thus, human fibroblasts proved to be much more potent producers of chemokines than colon epithelial cells. Such observation is reasonable in the context of these cells’ localization and function. Intestinal epithelium as main barrier has a contact with a broad spectrum of alien antigens, thus it should characterize by some level of tolerance not to potentiate immune response that could lead to pathological changes, e.g., IBD [31,32]. While the main role of fibroblasts in skin barrier is replacement tissue formation and wound closure, which must be accompanied by inflammatory response to keep wound clean [40,42].

HFF-1, similar to Caco-2, were most strongly stimulated by TNF to IL-8 production after just a short exposure (Figure 3a, Table 5). Equally strong stimulation was caused by ursolic acid (KU) after 24-h treatment of HFF-1 (Figure 3b, Table 5). Interestingly, all reference compounds were able to increase IL-8 production after long-lasting exposure although they had no such activity during first 6 h of their action. Even quercetin (Q), significantly inhibiting IL-8 production by HFF-1 for 6 h, stimulated the secretion of this chemokine after 24-h cell exposure (Figure 3, Table 5). Moreover, general mode of action of fractionated *E*. *rhamnoides* extracts in terms of IL-8 production on HFF-1 was completely opposed than their action on Caco-2: stimulatory effect on fibroblasts (Figure 3, Table 5) versus inhibitory effect on colon epithelial cells (Figure 1, Table 4).

Therefore, it is obvious, that not only chemical composition of plant-origin complex products determines their activity, but also the type of target cells. Cell functions determining the expression of surface receptors and also membrane permeability may play a key role in this process. Therefore, we would like to emphasize that it is very important to point target tissue for each natural product tested, when its biological activity is assessed and described. Also at least in vitro/ex vivo tests on cell culture are necessary, however in vivo study on animal models seems to be desirable too, to exclude any possible adverse effect in other tissues than these indicated as the target.

**Figure 4:**
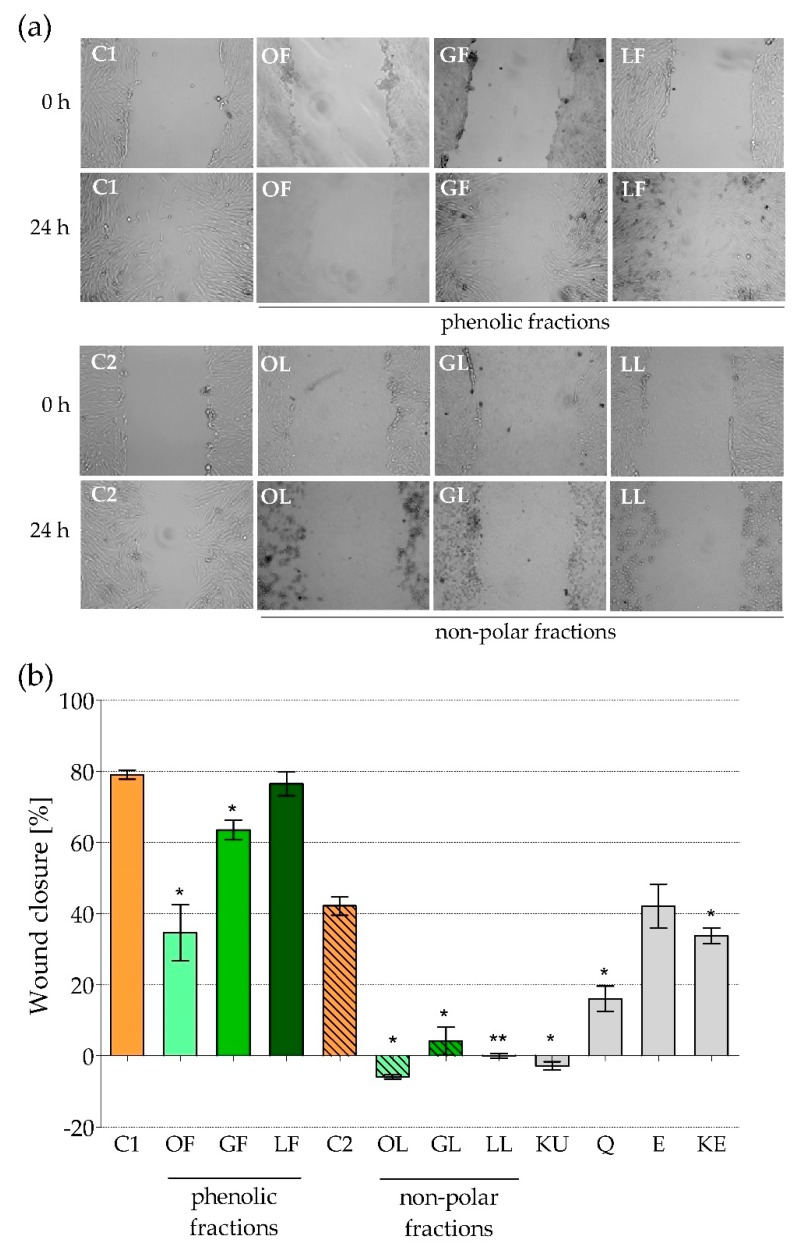
Human foreskin fibroblasts line HFF-1 migration after 24-h exposure to fractionated *E*. *rhamnoides* (L.) extracts and reference compounds, tested by scratch assay: (**a**) Representative pictures of the scars just after scratch (t0) and after 24 h cells exposure (t24), magnification 100×; (**b**) “wounds” closure given as a mean percentage ± standard error calculated based on scars area at t0 and t24. C1—control cells in culture medium with Me-OH, OF/GF/LF—phenolic fractions of fruit/twig/leaf extracts, C2—control cells in culture medium with DMSO, OL/GL/LL—non-polar fractions of fruit/twig/leaf extracts, KU—ursolic acid, Q—quercetin, E—(−)-epicatechin, KE—ellagic acid; * *p* < 0.05, ** *p* < 0.01.

We did not use animal model in presented studies, however encouraged by positive stimulatory activity of phenolic and non-polar fractions of *E*. *rhamnoides* extracts on fibroblasts chemokine secretion, we decided to check their effect on HFF-1 migration using in vitro scratch assay, which was supposed to simulate wound healing in vivo. In 24-h-old monolayers of the fibroblasts the scratches were done, which then were photographed and measured. Next the cells were exposed for 24 h to sea buckthorn extracts or reference compounds and then the appearance and surface area of the scars were assessed once again to estimate migration of HFF-1. Obtained results were surprisingly unfavorable. As it was shown in Figure 4 only phenolic fraction of *E*. *rhamnoides* leave extract (LF) did not have any effect on fibroblasts migration (the percentage of scratch closure reached 76.5 ± 3.4% in comparison to 79.1 ± 1.3% in control C1 cells; Figure 4b), while all other extracts significantly inhibited that process. Particularly strong inhibitory effect was observed for non-polar fractions, which completely stopped “wounds” closure (the areas of the “wounds” after 24-h cell treatment were exactly the same or even bigger than just after the scratch; Figure 4). The negative values of “wound” closure obtained for non-polar fraction of sea buckthorn fruit extract (OL; mean value: −5.9 ± 0.6%) and ursolic acid (KU; mean value: −2.8 ± 1.2%) points to detachment of the fibroblasts from bottom of the plates during 24 h of co-incubation and thus slight cytotoxic activity of OL and KU against HFF-1 cells. Of course, the impact of DMSO used as a solvent for non-polar fractions on cell migration was excluded by preparing appropriate control C2 cells in culture medium with DMSO, in which “wound” closure was lower than in C1, but still reached 42.2 ± 2.6% (Figure 4b). Most reference compounds tested caused the same inhibitory effect, except (−)-epicatechin (E), which did not affect fibroblasts migration comparing to control C2 cells (Figure 4b).

Obtained results do not fit to general image created in the literature in terms of positive activity of plant-origin extract on wounds healing. Many of medicinal plants act as wound healers, which has been confirmed in both in vitro and in vivo models. One of the best recognized and used since as early as 1500 B.C. to treat skin infections, burns, insect bites and eczemas is *Aloe vera* [43,44]. Antioxidant and anti-inflammatory activity of *Aloe vera* and its major components (aloesin, aloin, and emodin) have been accepted as the crucial pro-health mechanisms [44]. According to literature data, both types of the cells: Fibroblasts and keratinocytes, as well as their physiological activity important for wound healing are regulated by aloe-containing products. It was demonstrated, that such products increased the secretion of fibroblast growth factors, which later control the proliferation, transformation and secretory activity of these cells. Keratinocyte proliferation, migration and differentiation were also accelerated in the presence of aloe-containing preparations [45,46,47]. Aloe leaf extracts and gels improved wound healing also at in vivo models by a promotion of inflammatory cell infiltration, angiogenesis, extracellular matrix deposition and epithelialization [48]. A lot of other plants and derived products expressed similar effects supporting wound healing [41,43,46,49,50,51]. Upathyay et al. [52] demonstrated that aqueous leaf extract of sea buckthorn increased collagen synthesis and stabilization at the wound site, promote angiogenesis and through antioxidative activity decreased lipid peroxide levels in burn wound granulation tissue. Thus we also expected such a positive effect of well phytochemically characterized phenolic and non-polar fractions of the extracts from *E*. *rhamnoides* fruits, leaves and twigs. However, based on the results of our in vitro study we can suggest their using only on undamaged skin as the preparations supporting natural barrier by increasing of IL-8 production and thus innate immune cells recruitment. While, the application of fractionated sea buckthorn extracts on wounded skin should not to be advised, because of strong inhibitory impact on fibroblast migration.

To sum up presented research briefly, it was demonstrated for the first time, to the best of our knowledge, that both phenolic and non-polar fractions of *E*. *rhamnoides* (L.) extracts can possibly improve intestinal and skin barrier in healthy individuals. The rationale for the suggestion is a demonstration that some of sea buckthorn products tested caused the increase of ICAM-1 expression on colon epithelial cells and intensification of IL-8 production by fibroblasts. The oral administration of these extracts could also be attractive for patients with IBD to reduce inflammation by inhibiting of immunocompetent cells chemotaxis. However, since a supportive activity of sea buckthorn extracts in wound healing using scratch assay was not confirmed, their applying should be limited to a healthy skin. Moreover, because the research were carried out on cell cultures in vitro, to verify our hypotheses in vivo study on animal models need to be considered in future.

## 3. Materials and Methods

### 3.1. Chemicals and Media

2,2′-azinobis-(3-ethylbenzothiazoline-6-sulfonic acid (ABTS; commercially available solution for colour development in ELISA), bovine serum albumin (BSA), ethylenediaminetetraacetic acid (EDTA), quercetin, sodium dodecyl sulfate (SDS; 1% *w*/*v* solution used for stopping of color reaction in ELISA), Trizma base (a component of Tris-buffered Saline (TBS; 20 mM Trizma base, 150 mM NaCI) used as a basis to prepare dilution buffer in ELISA: TBS supplemented with 0.1% BSA and 0.05% Tween 20), trypan blue, Tween 20 (used to prepare wash buffer (0.05% Tween 20 in PBS) and dilution buffer in ELISA) and ursolic acid were purchased from Merck (Kenilworth, NJ, USA). Ellagic acid and (−)-epicatechin were obtained from Carl Roth (Karlsruhe, Germany). Dimethyl sulfoxide (DMSO) and methanol (Me-OH) were obtained from Chempur (Piekary Śląskie, Poland). Potassium chloride and sodium chloride were purchased from Standard Sp. z o.o. (Lublin, Poland). Disodium hydrogen phosphate and potassium dihydrogen phosphate were purchased from POCH (Gliwice, Poland).

Dulbecco’s Modified Eagle Medium high glucose with L-glutamine and sodium pyruvate (DMEM hg w/L-glu/sp), Modified Eagle Medium high glucose with L-glutamine and without sodium pyruvate (DMEM hg w/L-glu), Dulbecco’s Phosphate Bufferd Saline without calcium and magnesium (DPBS) and penicillin-streptomycin solution were purchased from Biowest (Nuaillé, France). Trypsin/EDTA solution and fetal bovine serum (FBS) were purchased from Biological Industries (Cromwell, CT, USA).

Human IL-8/CXCL8 DuoSet ELISA, human TNF-α DuoSet ELISA, murine TNF-α (TNF), murine monoclonal anti-human CCL-3/MIP-1 DuoSet ELISA, human ICAM-1/CD54 fluorescein-conjugated antibodies, rat monoclonal anti-human/mouse/bovine integrin alpha 6/CD49f phycoerythrin-conjugated antibodies and murine monoclonal IgG1 fluorescein-conjugated antibodies were purchased from R&D Systems (Minneapolis, MN, USA). Peptidoglycan from *S*. *aureus* (PG) was obtained from Sigma-Aldrich/Fluka (product brand) (Buchs/Schweiz, Germany).

### 3.2. Plant Material

Whole branches of sea buckthorn (*Elaeagnus rhamnoides* (L.) A. Nelson) were obtained from a horticultural farm in Sokółka, Podlaskie Voivodeship, Poland (53°24′N, 23°30′E). A voucher specimen (IUNG/HRH/2015/2) has been deposited at the Department of Biochemistry and Crop Quality, Institute of Soil Science and Plant Cultivation State Research Institute, Puławy, Poland. Fruit and leaves were hand-picked in the laboratory. Twigs were cut into pieces, dried in a laboratory drying chamber at 40 °C, and ground in laboratory mills (SM300, ZM200; Retsch, Haan, Germany). Freshly collected fruit and leaves were frozen. The leaves were subsequently freeze-dried (Gamma 2-16 LSC, Christ; Osterode am Harz, Germany) and milled in a laboratory mill (ZM200 Retsch). The fruit was ground frozen in a meat grinder, and freeze-dried. The freeze-dried fruit was defatted with hexane in a Soxhlet extractor. The dried and ground plant material was stored in a freezer.

### 3.3. Preparation of Fractionated Sea Buckthorn Extracts and Phytochemical LC-MS Analyses

Phenolic and non-polar fractions of sea buckthorn fruit, leaves and twigs were prepared as described by Olas et al. [21] and Różalska et al. (supplementary material) [20]. Briefly, the milled leaves (284 g), twigs (680 g) and fruit (1835 g) were extracted with 5 L (in 3 portions), 14 L (in 3 portions), and 27 L (in 2 portions) of 80% methanol (*v*/*v*), respectively, at room temperature. The extraction was additionally enhanced by short (10 min) cycles of sonication in an ultrasonic bath. Filtered extracts were concentrated by rotary evaporation and defatted with hexane. After removal of organic solvents in a rotary evaporator (Heidolph Instruments, Schwabach, Germany), the extracts were resuspended in MilliQ water and subjected to extraction with *n*-butanol. The butanol extracts were rotary evaporated, solid residues were mixed with MilliQ water or 20% *tert*-butanol, frozen, and freeze-dried. The butanol extract of sea buckthorn fruit was additionally subjected to C18 SPE on a short column (Cosmosil 140C18-PREP), to remove significant amounts of organic acids and sugars. Bound or sedimented compounds were eluted with 85% methanol (*v*/*v*), the eluate was concentrated in a rotary evaporator and freeze-dried. We obtained 12.42 g of the leaf extract, 24.64 g of the twig extract, and 24.94 g of the fruit extract. Each lyophilized butanol extract was subsequently dissolved (2 g per 100 mL) in 50% methanol (*v*/*v*), sonicated (2 min) and centrifuged. Supernatants were rotary evaporated to remove the organic solvent and freeze-dried, to yield phenolic-rich fractions. Pellets, which consisted mainly of hydrophobic compounds, were dissolved in methanol, and the solutions were rotary-evaporated to dryness. Solid residues were dissolved in mixtures of water and *tert*-butanol, and freeze dried. The phenolic fraction constituted 94.8%, 94.0%, and 79.1% of the sea buckthorn leaf, twig, and fruit extracts, respectively; the non-polar fraction constituted 5.2% of the leaf, 6.0% of the twig, and 20.9% of the fruit extract.

Composition of the investigated fractions was determined by UHPLC-ESI-HRMS/MS (Dionex UltiMate 3000 RSLC, Thermo Fisher Scientific, Waltham, MA, USA; Impact II, Bruker, Billerica, MA, USA). Detailed descriptions of chromatographic conditions and MS settings were published by Olas et al. [21] and Różalska et al. (supplementary materials) [20]. Constituents of the analyzed fractions were identified on the basis of their MS and UV spectra, with help of literature data. The relative content of individual compounds was determined on the basis of CAD chromatograms and expressed as a percentage of the total peak area. The composition of the sea buckthorn fractions was described and discussed in [21] and the supplementary material of [20], while recent updates can be found in [36,53]. The composition of the investigated fraction is also briefly shown in Table 1, Table 2 and Table 3.

### 3.4. Preparation of the Solutions of Fractionated Sea Buckthorn Extracts and Reference Compounds

Stock solutions of fractionated sea buckthorn extracts (8 mg/mL) were prepared in 100% methanol (phenolic fractions from fruits (OF), twigs (GF), and leaves (LF)) or 100% DMSO (non-polar fractions from fruits (OL), twigs (GL), and leaves (LL)), and further were diluted in a proper culture medium to obtain concentration required for a given experiment. Reference compounds ((−)-epicatechin (E), ellagic acid (KE), quercetin (Q), and ursolic acid (KU)) were dissolved in 100% DMSO and diluted in the appropriate medium. In selected tests the cells were additionally stimulated with PG and TNF-α, which stock solutions were prepared in DPBS.

### 3.5. Cell Cultures

Human epithelial colorectal adenocarcinoma cells (Caco-2, HTB-37) and human foreskin fibroblasts (HFF-1, SCRC-1041) were obtained from LGC Standards (Teddington, Middlesex, UK). Caco-2 cells were cultured according to manufacturer’s instruction in DMEM hg w/L-glu medium supplemented with 10% *v*/*v* FBS and penicillin-streptomycin solution at 37 °C in a humidified atmosphere of 5% CO_2_. HFF-1 cells were cultured in DMEM hg w/L-glu/sp medium supplemented with 15% *v*/*v* FBS and penicillin-streptomycin solution at 37 °C in a humidified atmosphere of 5% CO_2_.

### 3.6. ELISA Assay for Assessment of IL-8, MIP-1α, and TNF-α Production

Caco-2 or HFF-1 cells in the exponential growth phase were seeded (1 × 10^6^/well) on 24-well plates (Nunc, Roskilde, Denmark) and cultured for 24 h at 37 °C in a humidified atmosphere of 5% CO_2_. Then, the standard culture medium was replaced with a fresh medium containing 1.25% *v*/*v* Me-OH/DMSO (control cells C1/C2, respectively) or medium supplemented with *E*. *rhamnoides*-derived extracts (100 µg/mL), reference compounds (E, KE, Q, KU; 100 µg/mL), PG (1 µg/mL) or TNF (500 ng/mL) and the cells were cultured for 8 or 24 h at 37 °C in a humidified atmosphere of 5% CO_2_. After that time, the cell-free supernatants were collected and stored at −80 °C for further analysis. The assessment of IL-8, MIP-1α and TNF-α presence in cell-free supernatants were performed using commercial available DuoSet ELISA kits (R&D Systems, Minneapolis, MN, USA) according to manufacturer’s instructions.

### 3.7. Scratch Assay to Determine of the Fibroblasts Migration

HFF-1 cells in the exponential growth phase were seeded (2.5 × 10^5^/well) on 24-well plates with previously marked reference lines on the center of bottom of plate’s wells and allowed to grow to 70%–80% confluence as a monolayer. After 24 h the monolayer was gently scratched across the center of the wells with a sterile 200-µL pipette tip. The scratch was performed in a perpendicular way to the reference line, creating a cross in each well. After scratching, the medium with detached cells was removed, and the wells were washed twice in culture medium. Fresh medium containing 1.25% *v*/*v* Me-OH/DMSO (control cells C1/C2, respectively) or medium supplemented with fractionated sea buckthorn extracts (100 µg/mL) or reference compounds (E, KE, Q, KU; 100 µg/mL) was added to each well, and the cells were grown for 24 h. Images were obtained from the same fields immediately after scratching (t0) and then after 24 h (t24) using Motic Microscope model AE-2000T with inverted field of view and integrated camera (Conbest Sp. z o.o., Kraków, Poland). Images were analyzed using ImageJ software (National Institutes of Health (NIH), Bethesda, MD, USA) by manually selecting the wound region and recording the total area in pixels. Untreated scratched cells represented the control. The percentage of wound closure was calculated using the following formula:

(1) [(wound area t0− wound area t24)/wound area t0]×100

### 3.8. Assessment of ICAM-1 Expression on Caco-2 Cells Using Flow Cytometry

Caco-2 in the exponential growth phase were seeded (2.5 × 10^5^/well) on 24-well plates and cultured for 24 h at 37 °C in a humidified atmosphere of 5% CO_2_. Then, the standard culture medium was replaced with fresh medium containing 1.25% *v*/*v* Me-OH/DMSO (control cells C1/C2, respectively) or medium supplemented with tested extracts (100 µg/mL), reference compounds (E, KE, Q, KU; 100 µg/mL), PG (1 µg/mL) or TNF (500 ng/mL) and cells were cultured for 24 h at 37 °C in a humidified atmosphere of 5% CO_2_. After 24 h the culture medium was removed and the monolayer was gently washed twice with sterile DPBS and cells were detached with 15 mM EDTA/0.5% BSA solution in DPBS (10 min, on ice). The free-cell suspensions were transferred to eppendorf tubes and centrifuged (5 min, 2000 rpm, 4 °C). Obtained cell pellets were resuspended in 150 µL of staining buffer (0.5% BSA/0.1% sodium azide in DPBS) and then 50 µL aliquots were transferred to cytometric tubes, where previously 3 µL of antibodies (anti-CD49f/PE and anti-ICAM-1/FITC or anti-CD49f/PE and IgG1/FITC) were added. All samples were incubated on ice, in a dark for 30 min, washed twice with staining buffer and finally resuspended in the same buffer. Immediately all samples were measured on BD LSRII flow cytometer (Becton Dickinson, Franklin Lakes, NJ, USA) and analyzed with BD FACSDIVA software (Becton Dickinson, Franklin Lakes, NJ, USA). The expression of ICAM-1 on Caco-2 surface was measured on CD49f-positive cells (10^4^ cells/sample) from population of singlet cells (FSC-A vs. FSC-H analysis). The marker of FITC fluorescence was setup based on isotype control staining. The scheme of gating strategy is presented in Figure 2a.

### 3.9. Statistical Analysis

Student’s *t*-test, one-way ANOVA with Bonferroni correction and paired two-samples Wilcoxon test were calculated using STATISTICA 12.0 (StatSoft Polska Sp. z o.o., Kraków, Poland) software, with *p* ≤ 0.05 considered as significant. Data are mostly given as the means with standard error.

## 4. Conclusions

Despite the undoubted health-promoting properties of many plant-derived products, they cannot be considered universal “medicine”. Their activity, and thus pharmaceutical utility, depends on many different factors, such as the complex composition, the type of target cells, as well as the health and immune status of the host organism. The phenolic and non-polar fractions of *Elaeagnus rhamnoides* (L.) fruit, leaf, and twig extracts tested here are the best example. Based on their immunomodulatory activity found in our in vitro studies, only non-polar fractions of sea buckthorn twig and leaf extracts could theoretically be recommended as dietary supplements to improve the effectiveness of the intestinal barrier in healthy people. This suggestion is supported by their ability to induce of cellular ICAM-1 receptor expression, and in relation to the non-polar fraction of twig extract, additionally to enhance IL-8 production. In contrast, other types of fractionated extracts, acting as inhibitors of IL-8 secretion, may be proposed for oral intake by patients suffering from IBD. On the other hand, the demonstrated adverse effect of fractionated sea buckthorn extracts on human fibroblasts, limits their therapeutic potential for topical use only on healthy, unharmed skin. Due to actual usefulness in the food, pharmaceutical and cosmetics industry of *E*. *rhamnoides* fruits, seeds, and leaves (but not twigs) so far, our above suggestions concern mainly the fractions of fruit and leaf extracts. However, the results obtained for sea buckthorn twig extract could become a basis for potential medicinal or industrial application of this waste material in the future.

Although the presented hypotheses need to be confirmed in experimental animal models, the conducted research clearly indicates that more attention should be paid to the exact description of the activity of plant extracts in order to give appropriate recommendations for their safe use.

## Figures and Tables

**Table 1:** The relative content of individual groups of the compounds in phenolic fraction and non-polar fraction of sea buckthorn fruit extract, expressed as a percentage of the total peak area (Charged Aerosol Detector).

Group of Compounds	Relative Peak Area (%)	Dominant Compounds
phenolic fraction of the fruits (OF)		
Flavonol glycosides	67.5	I-3-*O*-Rut *, I-3-*O*-Glc *, I-3-*O*-Glc-7-*O*-Rha *, Q-3-*O*-Glc *, rutin *, I-3-*O*-Glc-7-*O*-(3′-*O*-IvA)-Rha
Other polar	21.0	unidentified, hexose, quinic acid/isomer
Triterpenoids and acylated triterpenoids	9.1	C_30_H_48_O_5_, C_30_H_48_O_6_
Other non-polar	2.4	unidentified
non-polar fraction of the fruits (OL)		
Flavonol glycosides	0.9	as in the phenolic fraction
Other polar	0.1	unidentified
Triterpenoids and acylated triterpenoids	83.6	oleanolic acid *, ursolic acid *, C_30_H_48_O_5_, C_30_H_48_O_4_, C_30_H_48_O_5_-pCouA, C_30_H_48_O_4_-pCouA, C_30_H_48_O_6_-pCouA
Other non-polar	15.4	unidentified

I—isorhamnetin; Q—quercetin; Glc—glucose; Rha—rhamnose; Rut—rutinoside; Hex—hexose; dHex—deoxyhexose; CouA—coumaric acid; IvA—isovaleric acid; *—confirmed using authentic standards.

**Table 2:** The relative content of individual groups of the compounds in phenolic fraction and non-polar fraction of sea buckthorn twig extract, expressed as a percentage of the total peak area (Charged Aerosol Detector).

Group of Compounds	Relative Peak Area (%)	Dominant Compounds
phenolic fraction of the twigs (GF)		
Proanthocyanidins and catechin	54.3	catechin, (epi)C-(epi)C, (epi)C-(epi)C-(epi)C, (epi)C-(epi)C-(epi)C-(epi)C, (epi)GC, (epi)C-(epi)GC
Flavonol glycosides	2.3	I-3-*O*-Glu-7-*O*-Rha *, I-3-*O*-Rut *, K-Hex-CouA
Hydrolysable tannins and ellagic acid	1.9	ellagic acid, casuarinin/isomer
Other polar	19.8	dihexose, quinic acid/isomer, unidentified
Spermidine derivatives	10.7	tricoumaroyl spermidine, feruloyl dicoumaroyl spermidine, diferuloyl coumaroyl spermidine, triferuloyl spermidine
Triterpenoids and acylated triterpenoids	6.7	C_30_H_48_O_5_, C_30_H_48_O_4_
Other non-polar	4.3	unidentified
non-polar fraction of the twigs (GL)		
Proanthocyanidins and catechin	1.3	catechin, (epi)C-(epi)C, (epi)C-(epi)C-(epi)C
Other polar	1.8	unidentified
Spermidine derivatives	2.0	as in the phenolic fraction
Triterpenoids and acylated triterpenoids	89.0	oleanolic acid *, ursolic acid *, C_30_H_48_O_5_, C_30_H_48_O_4_, C_30_H_48_O_5_-pCouA, C_30_H_48_O_4_-CafA
Other non-polar	5.9	unidentified

I—isorhamnetin; K—kaempferol; Q—quercetin; Glc—glucose; Rha—rhamnose; Rut—rutinoside; Hex—hexose; dHex—deoxyhexose; CouA—coumaric acid; CafA—caffeic acid; FerA—ferulic acid; LinA—(−)-linalool-1-oic acid/isomer (C_10_H_16_O_3_); (epi)C—(epi)catechin; (epi)GC—(epi)gallocatechin; *—confirmed using authentic standards.

**Table 3:** The relative content of individual groups of the compounds in phenolic fraction and non-polar fraction of sea buckthorn leaf extract, expressed as a percentage of the total peak area (Charged Aerosol Detector).

Group of Compounds	Relative Peak Area (%)	Dominant Compounds
phenolic fraction of the leaves (LF)		
Hydrolysable tannins and ellagic acid	31.3	casuarinin, casuarictin, strictinin, hippophaenin B or their isomers, ellagic acid
Flavonol glycosides	24.5	I-3-*O*-Glc-7-*O*-Rha *, I-Hex-dHex, I-3-*O*-Rut *, rutin *, Q-Hex, K-Hex-CouA, I-dHex-Hex-LinA
Other polar	17.4	dihexose, quinic acid/isomer, (epi)gallocatechin, unidentified
Triterpenoid saponins	15.0	C_71_H_112_O_31_, C_69_H_110_O_29_, C_65_H_102_O_27_, C_65_H_102_O_26_
Triterpenoids and acylated triterpenoids	7.6	C_30_H_48_O_5_, C_30_H_48_O_4_
Other non-polar	4.2	unidentified
non-polar fraction of the leaves (LL)		
Hydrolysable tannins	2.7	casuarinin, hippophaenin B or their isomers
Flavonol glycosides	2.6	As in the phenolic fraction
Other polar	1.2	dihexose, quinic acid/isomer, unidentified
Triterpenoid saponins	30.5	C_69_H_110_O_29_, C_63_H_100_O_25_, C_63_H_100_O_24_, C_57_H_90_O_20_
Triterpenoids and acylated triterpenoids	50.7	oleanolic acid *, ursolic acid *, C_30_H_48_O_5_, C_30_H_48_O_4_, C_30_H_48_O_5_-pCouA, C_30_H_48_O_4_-pCouA, C_30_H_48_O_5_-FerA
Other non-polar	12.3	unidentified

I—isorhamnetin; K—kaempferol; Q—quercetin; Glc—glucose; Rha—rhamnose; Rut—rutinoside; Hex—hexose; dHex—deoxyhexose; CouA—coumaric acid; CafA—caffeic acid; FerA—ferulic acid; LinA—(−)-linalool-1-oic acid/isomer (C_10_H_16_O_3_); (epi)C—(epi)catechin; (epi)GC—(epi)gallocatechin; *—confirmed using authentic standards.

**Table 4:** The mode of changes in IL-8 production by Caco-2 cells exposed 6 h and 24 h to fractionated *E*. *rhamnoides* (L.) extracts and reference compounds.

Preparation *	Action 6 h	Action 24 h	Control/Significance 6 h; 24 h
OF	↓ 1.3×	↓ 1.2×	C1/NS; NS
GF	↓ 2.3×	↓ 2.1×	C1/*p* = 0.0138; *p* = 0.0345
LF	↓ 3.5×	↓ 2.0×	C1/*p* = 0.0138; *p* = 0.0345
OL	↓ 2.2×	↓ 2.9×	C2/*p* = 0.0138; *p* = 0.0048
GL	↓ 1.5×	↑ 1.3×	C2/*p* = 0.0138; *p* = 0.0345
LL	↓ 1.4×	↓ 1.2×	C2/*p* = 0.0138; NS
PG	↑ 1.3×	↑ 1.7×	C2/*p* = 0.0138; *p* = 0.0345
TNF	↑ 12.5×	↑ 11.6×	C2/*p* = 0.0138; *p* = 0.0345
KU	↓ 1.2×	↑ 1.4×	C2/*p* = 0.0345; *p* = 0.0345
Q	↓ 2.7×	↓ 4.4×	C2/*p* = 0.0138; *p* = 0.0345
E	↓ 1.5×	↓ 1.3×	C2/*p* = 0.0138; *p* = 0.0345
KE	↓ 2.3×	↓ 1.1×	C2/*p* = 0.0138; NS

* Abbreviations as in legend to Figure 1; NS—no significant difference (*p* > 0.05). ↑: increase, ↓: decrease compared to control.

**Table 5:** The mode of changes in IL-8 production by HFF-1 fibroblasts exposed 6 h and 24 h to fractionated *E*. *rhamnoides* (L.) extracts and reference compounds.

Preparation *	Action 6 h	Action 24 h	Control/Significance 6 h; 24 h
OF	↑ 2.0×	↑ 2.0×	C1/*p* = 0.0138; *p* = 0.0138
GF	↓ 1.1×	↑ 1.8×	C1/*p* = 0.0138; *p* = 0.0138
LF	↑ 1.7×	↑ 1.5×	C1/NS; NS
OL	↑ 1.7×	↑ 2.6×	C2/*p* = 0.0138; *p* = 0.0133
GL	↓ 1.2×	↑ 1.2×	C2/NS; *p* = 0.0138
LL	↑ 2.0×	↑ 1.1×	C2/*p* = 0.0138; NS
PG	↑ 1.3×	↑ 1.8×	C2/*p* = 0.0138; *p* = 0.0138
TNF	↑ 4.8×	↑ 3.0×	C2/*p* = 0.009; *p* = 0.0138
KU	↓ 1.1×	↑ 3.2×	C2/NS; *p* = 0.0138
Q	↓ 3.2×	↑ 1.7×	C2/*p* = 0.0138; NS
E	no change	↑ 2.0×	C2/NS; *p* = 0.0138
KE	↑ 1.1×	↑ 2.1×	C2/*p* = 0.0138; NS

* Abbreviations as in legend to Figure 3; NS—no significant difference (*p* > 0.05). ↑: increase, ↓: decrease compared to control.
